# Echinococcosis: An Uncommon Cause of Thoracic Pain

**DOI:** 10.7759/cureus.73792

**Published:** 2024-11-16

**Authors:** Beatriz Sampaio, Felisbela Gomes, Mafalda Leal, Rita Bernardino

**Affiliations:** 1 Internal Medicine, Centro Hospitalar Universitário de Lisboa Central, Lisbon, PRT

**Keywords:** cystic echinococcosis, echinococcosis, echinococcus granulosus, hydatid cyst, hydatidosis

## Abstract

Echinococcosis is caused by larvae, the metacestode stage of the tapeworm *Echinococcus*, and poses a public health issue in many developing countries. It generally affects a single organ, most commonly the liver or lungs, and rarely involves multiple organs. We present the case of a 24-year-old Indian man living in Portugal, who was admitted to the Emergency Department with left-sided thoracic pain radiating to the back. Chest computed tomography (CT) revealed a cystic nodule in the lower left lung. Laboratory tests showed high C-reactive protein levels and positive antibodies for *Echinococcus granulosus*. He was started on albendazole and underwent cranial and abdominopelvic CT scans, which identified a similar lesion in the liver. He underwent surgical removal of the hepatic cyst, followed by lung resection a couple of months later. This case underscores the importance of an early and accurate diagnosis, especially in non-endemic areas where the disease is less common and knowledge about it is more limited. It also emphasizes the need for stricter screening protocols for individuals with a history of exposure to endemic areas, even in low-incidence regions like Lisbon, Portugal.

## Introduction

Echinococcosis, also known as hydatid disease, is a zoonotic disease caused by the larvae, which represent the metacestode stage of the tapeworm *Echinococcus* [1,2].

The majority of human infections are caused by *Echinococcus granulosus*, which is responsible for cystic echinococcosis (CE), typically affecting a single organ - most commonly the liver or lungs - and rarely involving multiple organs. Although human CE is a notifiable parasitic infectious disease in most European countries, it is largely under-reported by national health systems and is part of the current group of 20 neglected tropical diseases of global health significance, endorsed by the WHO for prevention and control [3-5].

The CE has a global distribution and presents significant public health and economic challenges in many countries [1,3]. However, there seems to be a general decrease in incidence rates, although trends vary by country. This decline may be attributed to improved hygiene, rural-to-urban migration, reductions in sheep populations, increased intensive farming, and the implementation of national control programs. In contrast, unexpected increases have been noted in most non-endemic northern and western European countries, as well as in southern Baltic countries. This trend (accounting for hundreds of cases) may be due to increased migration from endemic regions, international travel, and greater awareness among physicians. In Portugal, CE is currently a rare disease, with an annual incidence of cases between 1997 and 2020 of 0.19 per 100,000 inhabitants [3].

Hydatidosis can be asymptomatic but may also result in serious outcomes. Clinical symptoms vary significantly depending on several factors, including (a) the affected organ, (b) the size and location of the cyst, (c) interactions between the growing cyst and adjacent organs, and (d) complications arising from cyst rupture [6-9].

## Case presentation

**Figure 1:**
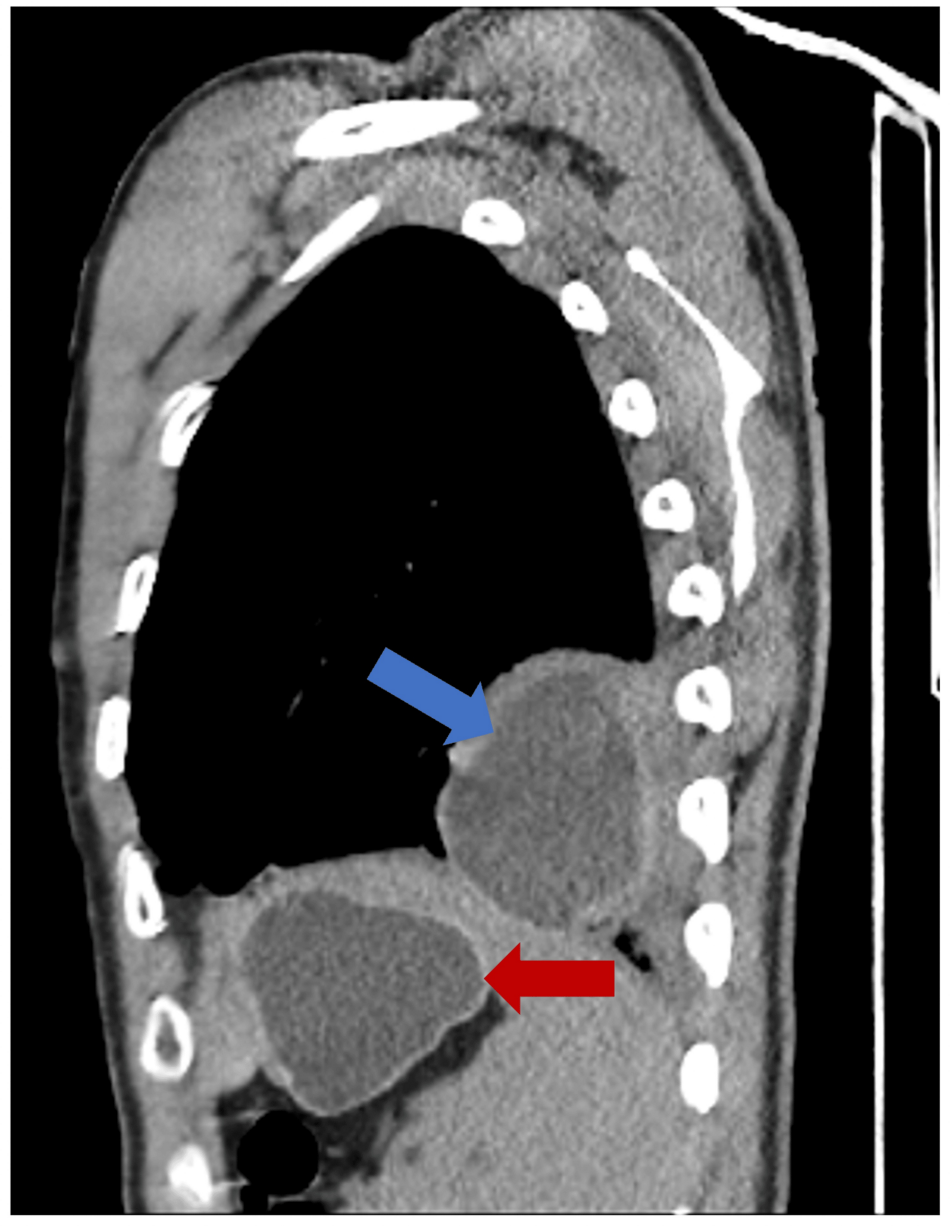
Thoracic and abdominal CT scan (sagittal view) revealing two hydatid cysts. The blue arrow shows a hydatid cyst in the lower lobe of the left lung; the red arrow shows a hydatid cyst in the left liver lobe. CT, computed tomography

**Figure 2:**
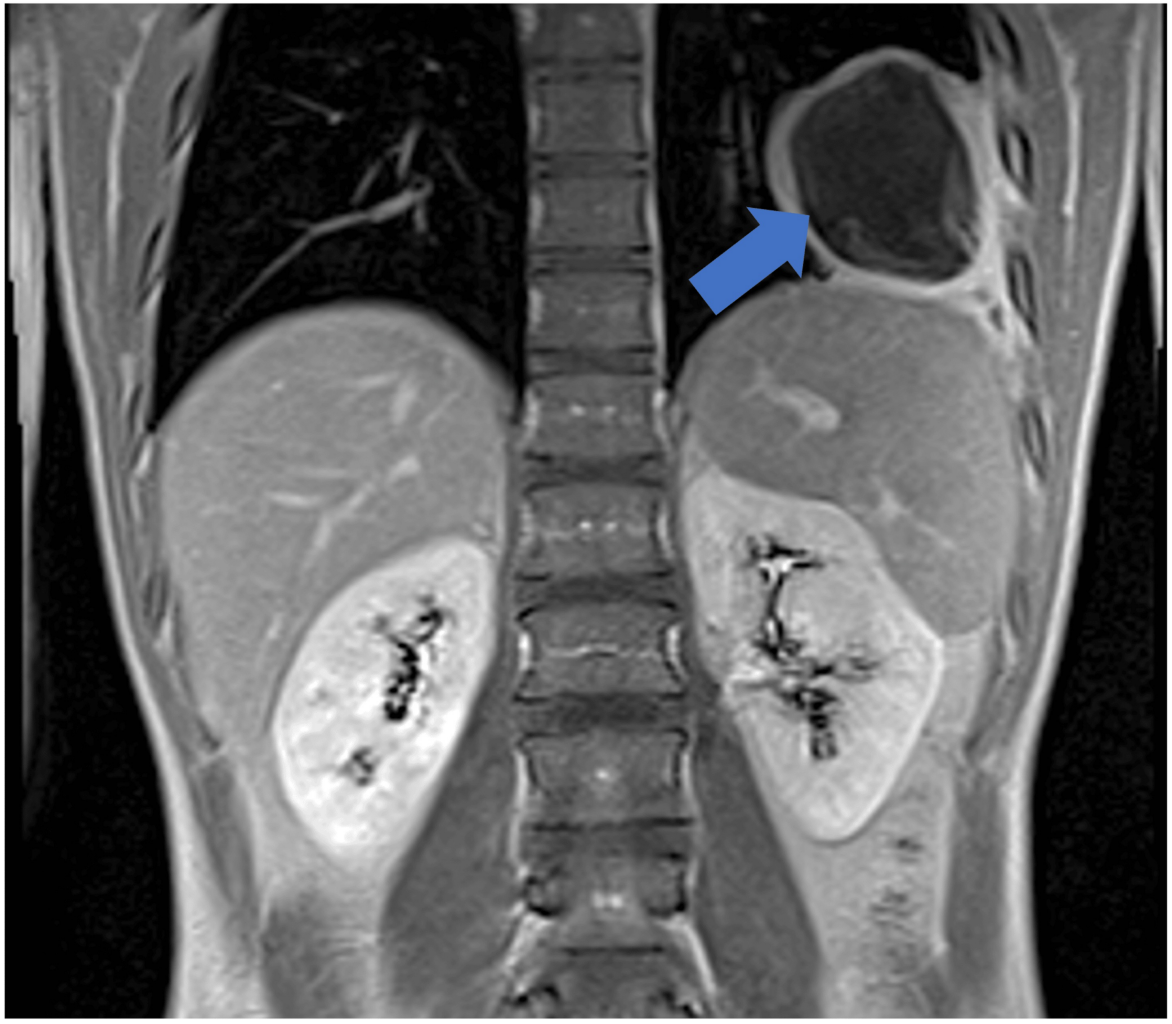
Thoracic and abdominal MRI (coronal view). The blue arrow shows a hydatid cyst in the left lung parenchyma. MRI, magnetic resonance imaging

**Figure 3:**
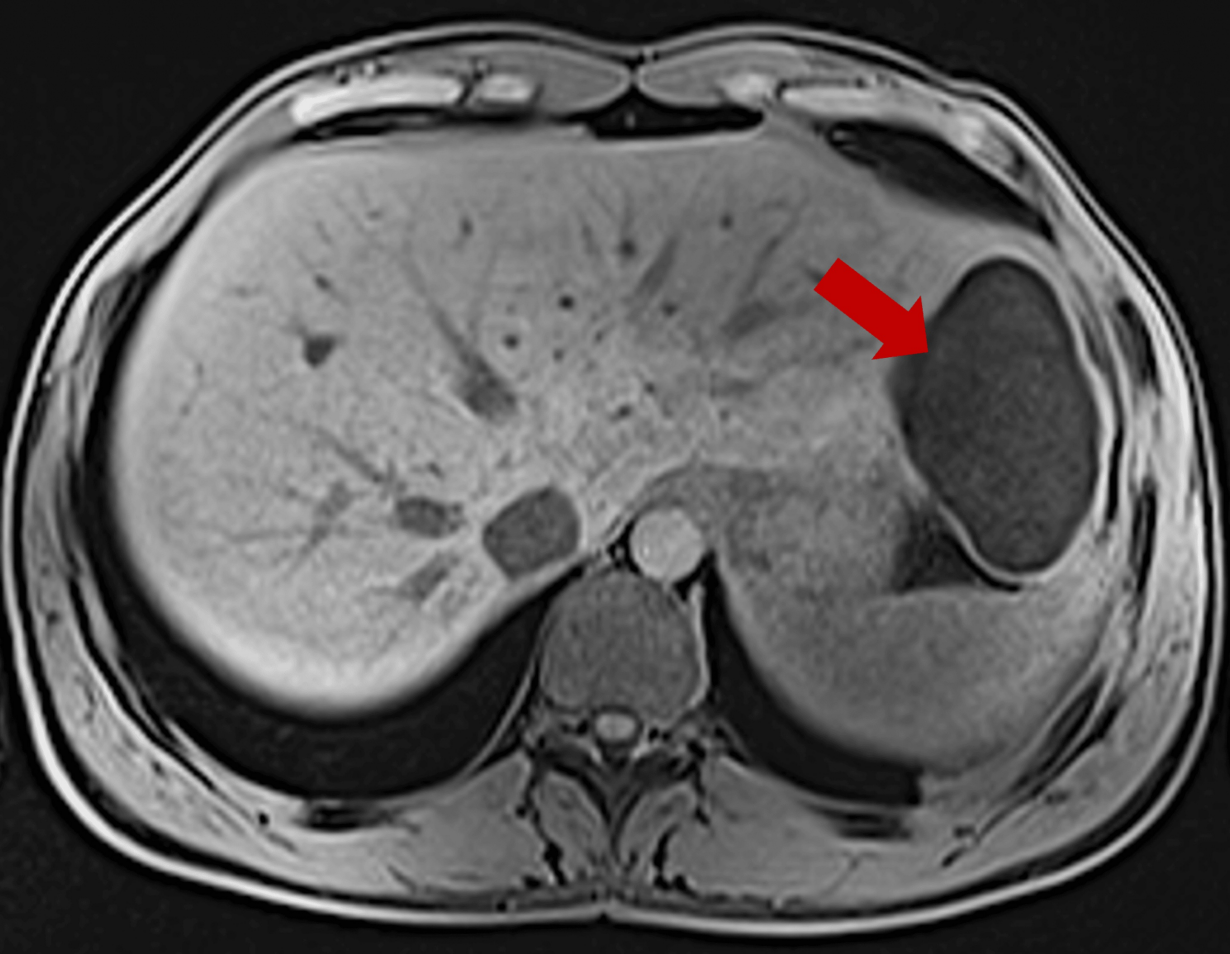
Abdominal MRI (axial view). The red arrow shows a hepatic hydatid cyst in the left lobe. MRI, magnetic resonance imaging

**Figure 4:**
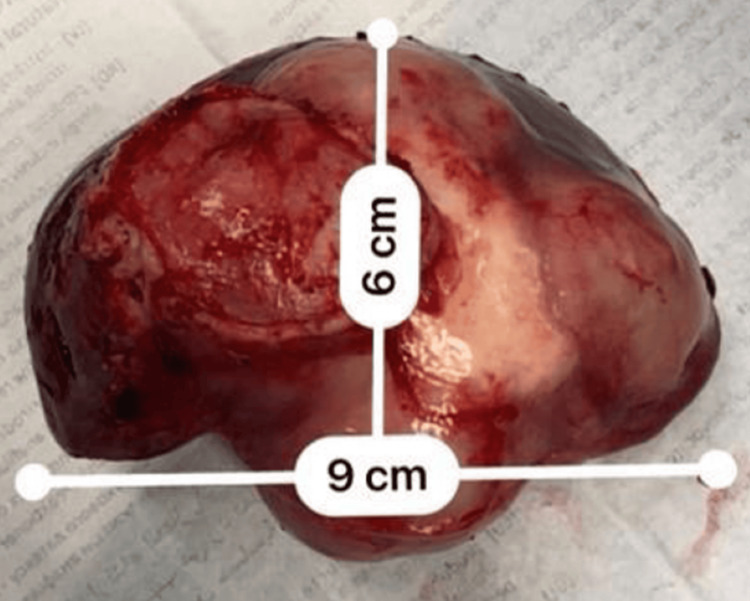
Macroscopic features of the resected lesion (hydatid cyst).

A 24-year-old Indian man, who has been living in Lisbon, Portugal, for three years and works for a company installing air conditioning, presented to the Emergency Department with three days of left-sided pleuritic chest pain radiating to the back. He had no relevant medical or surgical history and denied associated symptoms, such as dyspnea, cough, or fever. Clinical examination revealed decreased breath sounds at the left lung base on pulmonary auscultation. Chest radiography showed a nonspecific round opacity in the lower lobe of the left lung. A chest computed tomography (CT) scan was performed, revealing a unilocular cystic nodule measuring approximately 72 x 46 mm. The scan was then extended to the abdominopelvic region, identifying another cystic nodular formation (63 x 38 mm) with slightly thickened walls in the left liver lobe, consistent with a hydatid cyst (Figure 1). A cranial CT scan showed no lesions. Laboratory findings highlighted a C-reactive protein level of 97.5 mg/L and positive anti-*E. granulosus* antibodies with a titer of 1/640. The remaining blood tests were normal, and other serological studies were negative. He was started on albendazole 400 mg every 12 hours. A cranial magnetic resonance imaging (MRI) and a thoraco-abdominopelvic MRI (Figures 2-3) confirmed the findings described in the CT scans. Two weeks later, an elective laparoscopic resection of hepatic segments II/III was performed, removing a 9 x 6 x 5 cm lesion, histologically consistent with a hydatid cyst (Figure 4). He continued albendazole therapy and underwent a left lower lobectomy two months later, which was also confirmed histologically as a hydatid cyst. The patient was discharged without complications and instructed to continue albendazole therapy until the next evaluation but was subsequently lost to follow-up after immigrating to Switzerland.

## Discussion

Diagnosis of CE is guided by clinical suspicion, along with epidemiological history, clinical signs, imaging studies, and serological testing [10]. An early, accurate diagnosis, combined with prompt and appropriate treatment, is essential for better clinical outcomes and fewer post-surgical complications.

In non-endemic areas, diagnosis is particularly challenging due to the low prevalence of the disease and limited familiarity among healthcare professionals. Our patient had been living in Lisbon, a non-endemic area, for three years but is originally from India, an endemic region where the annual incidence varies from 1 to 200 per 100,000 inhabitants [11]. It is known that most primary infections typically occur in childhood, progressing through a subclinical phase with an incubation period of 5 to 15 years, often without early clinical symptoms [10].

This case highlights the importance of considering the epidemiological context to enable early diagnosis and treatment, leading to successful surgical management and extraction of hydatid cysts from the lung and liver. Therefore, in a patient originally from or previously residing in an endemic area, or with a possible history of exposure, who presents with respiratory or abdominal symptoms - especially when cysts are identified - echinococcosis should be considered a potential diagnosis. In most cases, including our patient's case, surgery combined with anti-parasitic therapy is generally the definitive treatment, aiming to prevent complications that can lead to larval seeding. While surgical intervention can be curative, cyst recurrence is common in the absence of anti-parasitic treatment. Patients are advised to undergo serial imaging over several years post-resection to monitor for refractory seeding and recurrence. This case underscores the significant risk associated with lack of follow-up, which may increase the likelihood of undetected recurrence and related complications [12].

The findings also emphasize the need for more aggressive screening protocols for patients with historical geographic or occupational risk factors, even in low-incidence regions like Lisbon.

## Conclusions

Future research should prioritize the development of enhanced preventive strategies, increased awareness, and improved medical record-keeping to optimize patient care in endemic regions. Regular screening is advisable for individuals residing in rural endemic areas, those engaged in high-risk occupations, and household members of infected patients, to enable early detection and intervention.
